# Two new species of oribatid mites of the family Galumnidae (Acari, Oribatida) from Vietnam

**DOI:** 10.3897/zookeys.382.6831

**Published:** 2014-02-20

**Authors:** Sergey G. Ermilov, Alexander E. Anichkin

**Affiliations:** 1 Tyumen State University, Tyumen, Russia; 2 Joint Russian-Vietnamese Tropical Research and Technological Center, Hanoi-Ho Chi Minh, Vietnam; 3 A.N. Severtsov Institute of Problems of Ecology and Evolution, Russian Academy of Sciences, Moscow, Russia

**Keywords:** Oribatida, Galumnidae, *Allogalumna*, *Galumna*, new species, key, *calcicola*-group, Vietnam, Oriental region

## Abstract

Two new species of oribatid mites of the family Galumnidae, *Allogalumna monodactyla* **sp. n.** and *Galumna* (*Galumna*) *paracalcicola* **sp. n.**, are described from dark loamy soil under crown of *Ficus* sp. in southern Vietnam. *Allogalumna monodactyla* **sp. n.** is the first identified member of *Allogalumna* recorded for Vietnam. The identification keys to the species of *Allogalumna* from the Oriental region and species of *Galumna* (*Galumna*) from Vietnam and the *calcicola*-group are given.

## Introduction

During taxonomic identification of oribatid mites from Dong Nai Biosphere Reserve of southern Vietnam, we found two new species of Galumnidae; one belonging to the genus *Allogalumna* Grandjean, 1936, other to *Galumna (Galumna)* Heyden, 1826. The main goal of this paper is to describe these species.

*Allogalumna* is a genus that was proposed by Grandjean (1936) with *Galumna alamellae* Jacot, 1935 as type species. Currently, it comprises more than 30 species having a cosmopolitan distribution collectively (data summarized by Subías (2004, updated 2013)). In the Vietnamese fauna, *Allogalumna* has been recorded earlier, but some unidentified species has been referred (Vu et al. 1985; Ermilov and Anichkin 2013a). Thus, the new species described here is the first identified member of this genus recorded for Vietnam. We compared our present material with that of previously found one specimen of *Allogalumna* sp. (Ermilov and Anichkin 2013a), and clarified that the latter was the same species.

*Galumna* is a genus that was proposed by Heyden (1826) with *Notaspis alatus* Hermann, 1804 as type species. Currently, it comprises seven subgenera and more than 180 species having a cosmopolitan distribution collectively (data summarized by Subías (2004, updated 2013)). Among those subgenera, *Galumna (Galumna)* is a largest subgenus, comprising about 160 species. At present, this subgenus represented by 10 species in the Vietnamese fauna (Golosova 1983; Mahunka 1989; Krivolutskiy et al. 1997; Ermilov and Anichkin 2010, 2011b, 2013a, 2013b, 2013c, 2013d; Ermilov and Vu 2012; Ermilov et al. 2012; Ermilov and Niedbała 2013).

The generic diagnoses of the genera *Allogalumna* and *Galumna* are summarized earlier by Ermilov et al. (2013b).

Additionally, the identification keys to the *Allogalumna*-species from the Oriental region and *Galumna (Galumna)*-species from Vietnam and the *calcicola*-group are given in the present work.

## Material and methods

Three specimens (holotype: female; two paratypes: female and male) of *Allogalumna monodactyla* sp. n. and two specimens (holotype and paratypes: both females) of *Galumna (Galumna) paracalcicola* sp. n. are from: southern Vietnam, 11°26'12"N, 107°24'59"E, Dong Nai Province, Dong Nai Biosphere Reserve, dark loamy soil under crown of large tree (about 40 m height) *Ficus* sp., 30.XI.2013 (collected by A.E. Anichkin and S.G. Ermilov).

Soil samples were collected by taking 10 soil-cores (diameter: 7.8 cm; depth: 10 cm). Samples were left in the metal cores to minimize disturbance during transport from the field to the laboratory. Mites were extracted into 75% ethanol using Berlese’s funnels with electric lamps (40 W) for ten days.

Holotypes and paratypes were mounted in lactic acid on temporary cavity slides for measurement and illustration. The body length was measured in lateral view, from the tip of the rostrum to the posterior edge of the ventral plate. The notogastral width refers to the maximum width in dorsal aspect (without pteromorphs). Lengths of body setae were measured in lateral aspect. All body measurements are presented in micrometers. Formulae for leg setation are given in parentheses according to the sequence trochanter–femur–genu–tibia–tarsus (famulus included). Formulae for leg solenidia are given in square brackets according to the sequence genu–tibia–tarsus. General terminology used in this paper follows that of F. Grandjean (summarized by Norton and Behan-Pelletier 2009).

## Descriptions of new species

### Allogalumna monodactyla

Ermilov & Anichkin sp. n.

http://zoobank.org/7E80EBC2-CED5-4D18-996C-A08195970871

http://species-id.net/wiki/Allogalumna_monodactyla

Figs 1 –6

#### Diagnosis.

Body size 180–188 × 114–123. Body and legs covered by the microgranular cerotegument. Rostral, lamellar and interlamellar setae minute; lamellar setae little longer. Sensilli with disk-like head, having seven cilia. Anterior notogastral margin not developed. Four pairs of porose areas small, rounded, punctiform. Median pore located in centrodorsal part of notogaster. Postanal porose area absent. Legs monodactylous.

#### Description.

*Measurements*. Small species. Body length: 188 (holotype), 180, 184 (two paratypes); notogaster width: 123 (holotype), 114, 118 (two paratypes).

*Integument*. Body color yellowish-brown to brown. Body and legs covered by the microgranular cerotegument. Granules (up to 1) visible only under high magnification. Body surface smooth. Pteromorphs with distinct radiate wrinkles.

*Prodorsum*. Rostrum widely rounded. Rostral (*ro*, 2), lamellar (*le*, 4) and interlamellar (*in*, 2) setae thin, smooth. Sensilli (*ss*, 24–28) with short stalk and well-developed disk-like head, having seven cilia (all inserted in one row) of medium size. Exobothridial setae absent. Sublamellar lines (*S*) distinct, typical for *Allogalumna*: long, curving backwards. Porose areas *Ad* not founded.

*Notogaster*. Anterior notogastral margin not developed. Dorsophragmata absent. Notogastral setae represented by 10 pairs of alveoli. Four pairs of porose areas small, round (*Aa*, 4–6; *A1*–*A3*, 4), poorly visible, punctiform, without distinct borders. Alveoli of setae *la* inserted latero-posteriorly to *Aa*. Lyrifissures *im* located between *lm* and *lp.* Opisthonotal gland openings not evident. Median pore (*mp*) present in all specimens, located in centrodorsal part of notogaster between the virtual lines connecting *lm* and *lm*, *lp* and *lp.*

*Gnathosoma*. Morphology of subcapitulum, palps and chelicerae typical for most Galumnidae (for example: Ermilov and Anichkin 2010, 2011a, 2013b; Ermilov et al. 2011, 2013a). Subcapitular setae *h* minute (4), thin, smooth.

*Epimeral and lateral podosomal regions*. Apodemes (1, 2 sejugal, 3) well visible. Four pairs of epimeral setae observed ventrally; *1a*, *3a*, *3b*, *4a* short (4), thin, smooth. Discidia (*dis*) triangular, circumpedal carinae (*cp*) distinct.

*Anogenital region*. Six pairs of genital (*g*_1_–*g*_6_), one pair of aggenital (*ag*), two pairs of anal (*an*_1_, *an*_2_) and three pairs of adanal (*ad*_1_–*ad*_3_) setae little differs in size, minute (3–4), thin, smooth. Anterior edge of genital plates with two setae. Adanal setae *ad*_3_ inserted laterally to adanal lyrifissures *iad.* Postanal porose area absent.

*Legs*. Monodactylous; claw of each leg smooth. Morphology of leg segments, setae and solenidia typical for most Galumnidae (for example: Ermilov and Anichkin 2010, 2011a, 2013b; Ermilov et al. 2010, 2011), but solenidion of genua III weakly dilated in medial part. Formulae of leg setation and solenidia: I (1–4–3–4–20) [1–2–2], II (1–4–3–4–15) [1–1–2], III (1–2–1–3–15) [1–1–0], IV (1–2–2–3–12) [0–1–0]; homology of setae and solenidia indicated in Table 1.

**Figures 1–4.**
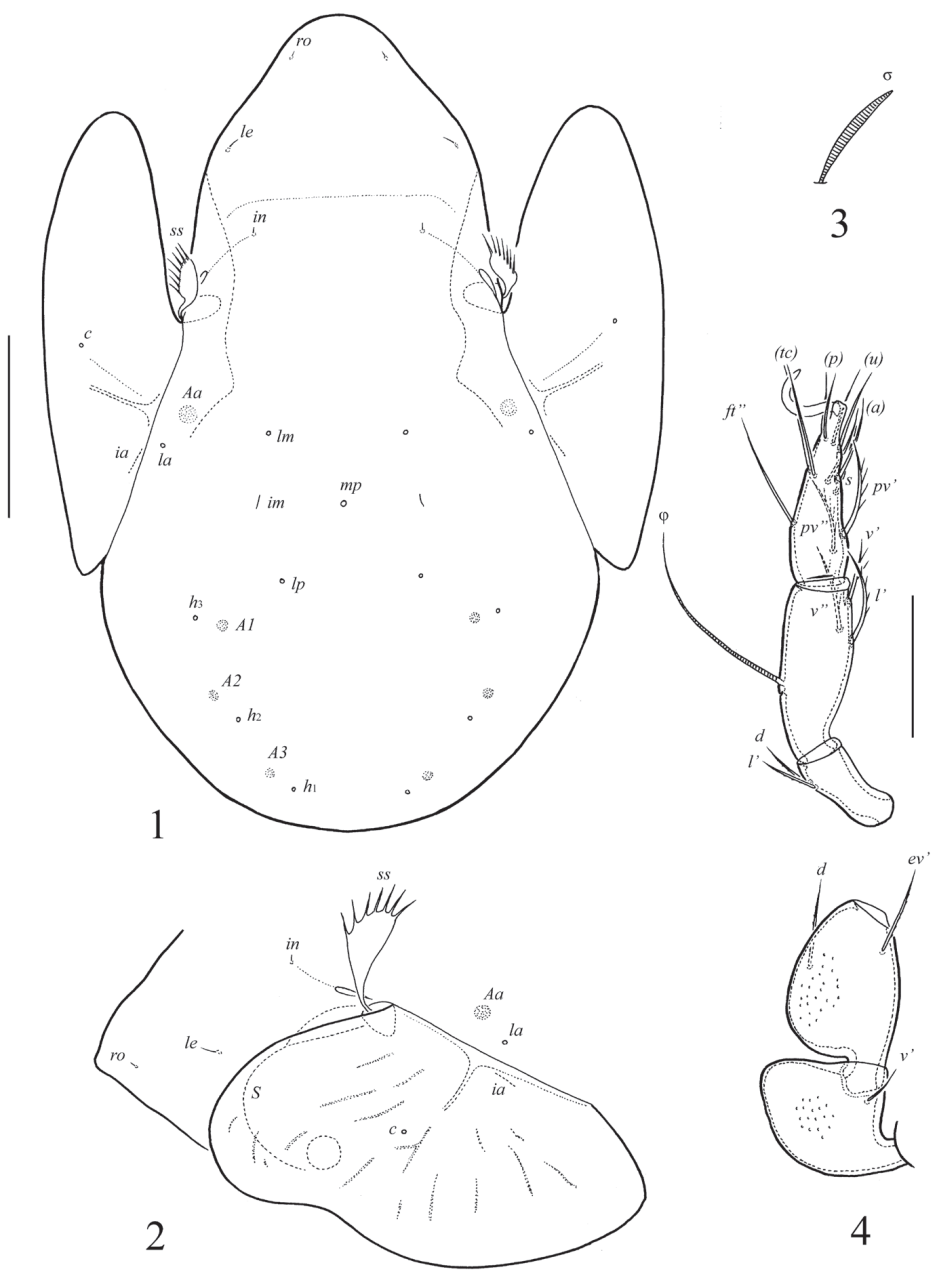
*Allogalumna monodactyla* sp. n., adult: **1** dorsal view **2** dorso-lateral view of prodorsum, pteromorph and anterior part of notogaster **3** solenidion of leg genu III **4** leg IV, left, antiaxial view. Scale bar (**1, 2**) 50 μm, (**3, 4**) 20 μm.

**Figures 5–6.**
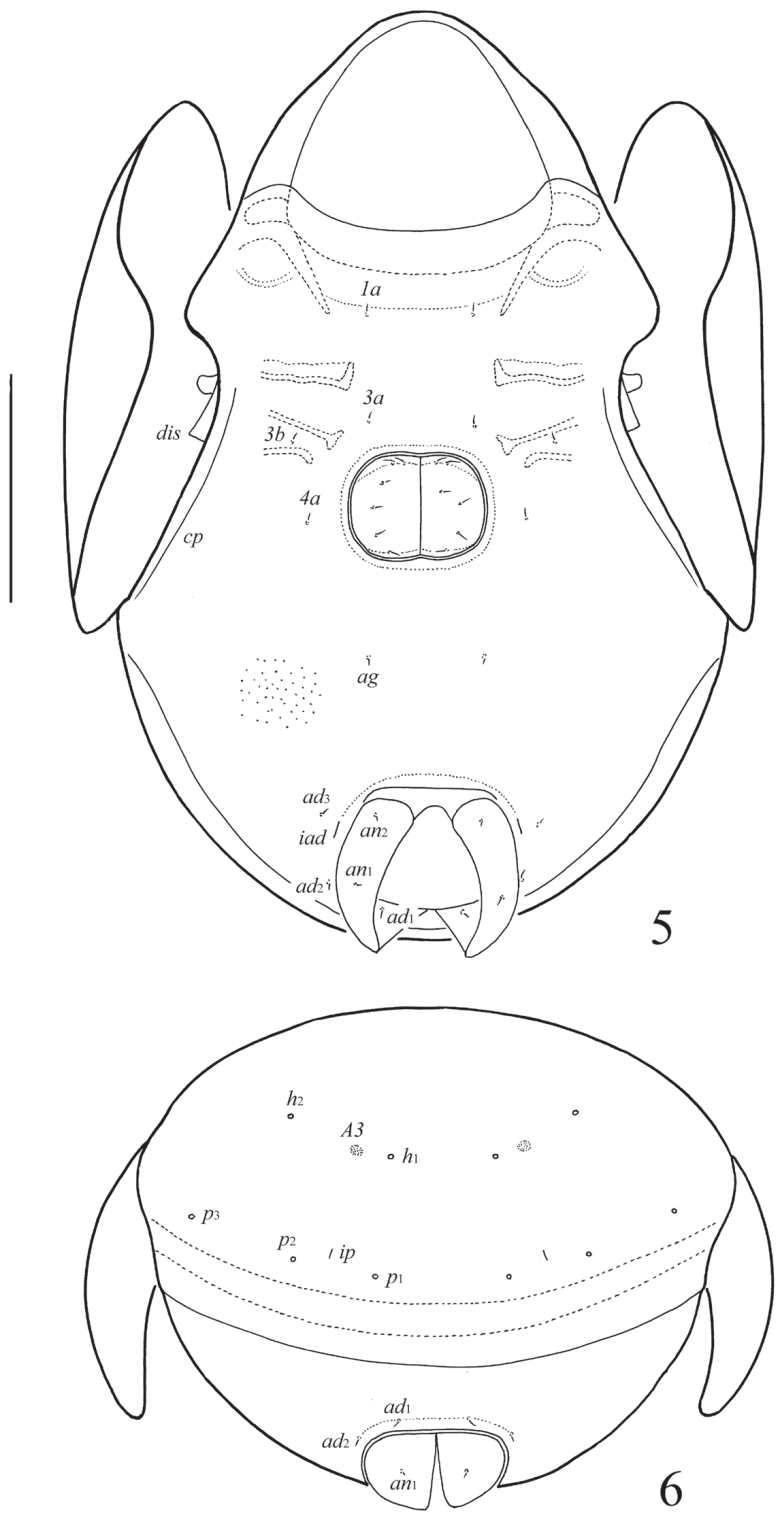
*Allogalumna monodactyla* sp. n., adult: **5** ventral view (gnathosoma and legs not illustrated) **6** posterior view. Scale bar 20 μm.

**Table 1.** Leg setation and solenidia of adult *Allogalumna monodactyla* sp. n. (same data for *Galumna (Galumna) paracalcicola* sp. n.).

Leg	Trochanter	Femur	Genu	Tibia	Tarsus
I	*v*’	*d*, (*l*), *bv*’’	(*l*), *v*’, **σ**	(*l*), (*v*), **φ**_1_, **φ**_2_	(*ft*), (*tc*), (*it*), (*p*), (*u*), (*a*), *s*, (*pv*), *v*’, (*pl*), *l*’’, *e*, **ω**_1_, **ω**_2_
II	*v*’	*d*, (*l*), *bv*’’	(*l*), *v*’, **σ**	(*l*), (*v*), **φ**	(*ft*), (*tc*), (*it*), (*p*), (*u*), (*a*), *s*, (*pv*), **ω**_1_, **ω**_2_
III	*v*’	*d*, *ev*’	*l*’, **σ**	*l*’, (*v*), **φ**	(*ft*), (*tc*), (*it*), (*p*), (*u*), (*a*), *s*, (*pv*)
IV	*v*’	*d*, *ev*’	*d*, *l*’	*l*’, (*v*), **φ**	*ft*’’, (*tc*), (*p*), (*u*), (*a*), *s*, (*pv*)

Roman letters refer to normal setae (*e* to famulus), Greek letters to solenidia. Single prime (’) marks setae on anterior and double prime (’’) setae on posterior side of the given leg segment. Parentheses refer to a pseudosymmetrical pair of setae.

#### Type deposition.

The holotype is deposited in the collection of the Zoological Institute of the Russian Academy of Sciences, St. Petersburg, Russia; one paratype in deposited in the collection of the Siberian Zoological Museum, Novosibirsk, Russia; one paratype is deposited in the collection of the Tyumen State University Museum of Zoology, Tyumen, Russia.

#### Etymology.

The specific name “*monodactyla*” refers to the monodactylous legs of the new species.

#### Comparison.

*Allogalumna monodactyla* sp. n. differs from other known species of the genus *Allogalumna* by the median pore located in centrodorsal part of notogaster (versus in posterior part) and monodactylous legs (versus tridactylous).

### Key to species *Allogalumna* of the Oriental region

**Table:** 

1	Sensilli with disk-like head, having seven cilia of medium size; median pore located in centrodorsal part of notogaster, legs monodactylous	*Allogalumna monodactyla* sp. n. (body size: 180–188 × 114–123; distribution: Vietnam)
–	Sensilli without disk-like head; median pore located in posterior part of notogaster, legs tridactylous	2
2	Rostrum pointed; anterior notogastral margin developed; three pairs of porose areas present	*Allogalumna gedaii* Mahunka, 1995 (body size: 449–505 × 312–346; distribution: Thailand; see Mahunka 1995)
–	Rostrum rounded; anterior notogastral margin not developed medially; four pairs of porose areas present	3
3	Sensilli setiform; anal plates striate longitudinally; only rostral setae present, and lamellar and interlamellar setae represented by alveoli	*Allogalumna asetosa* Ermilov & Kalúz, 2014 (body size: 564–581 × 415; distribution: India; see Ermilov and Kalúz 2014)
–	Sensilli with dilated head; anal plates not striate; all prodorsal setae present or represented by alveoli	4
4	Sensilli with long (longer than head), ciliate stalk; rostral, lamellar and interlamellar setae present; porose areas *Aa* similar to *A1*–*A3* in size	*Allogalumna incomplecta* Mahunka, 1988 (body size: 277–307 × 198–218; distribution: Borneo; see Mahunka 1988)
–	Sensilli with short (not longer than head), smooth stalk; rostral, lamellar and interlamellar setae represented by alveoli; porose areas *Aa* larger than *A1*–*A3*	*Allogalumna quadrimaculata* Mahunka, 1988 (body size: 389–405 × 275–300; distribution: Borneo; see Mahunka 1988)

### Galumna (Galumna) paracalcicola

Ermilov & Anichkin sp. n.

http://zoobank.org/86204514-8F54-4F36-80FA-558D69E90651

http://species-id.net/wiki/Galumna_paracalcicola

Figs 7 –10

#### Diagnosis.

Body size 398–415 × 298–332. Lamellar lines short, almost straight. Prodorsal setae long, setiform; rostral and lamellar setae slightly barbed, interlamellar setae smooth. Sensilli with long stalk and shorter, lanceolate, indistinctly barbed head. Anterior notogastral margin weakly developed. Four pairs of porose areas rounded. Median pore and postanal porose area present.

#### Description.

*Measurements*. Body of medium size. Body length: 398 (holotype), 415 (paratype); notogaster width: 298 (holotype), 332 (paratype).

*Integument*. Body color yellowish-brown. Body surface smooth, but some transverse stria located posteriorly to the genital apertures. Pteromorphs with distinct radiate wrinkles.

*Prodorsum*. Rostrum widely rounded. Rostral (61–65) and lamellar (73–77) setae setiform, weakly barbed. Interlamellar setae (102–110) setiform, smooth. Sensilli (86–90) with long stalk and shorter, lanceolate, indistinctly barbed head. Exobothridial setae absent. Sublamellar lines distinct, typical for *Galumna (Galumna)*: long, curving backwards. Lamellar lines (*L*) specific: rather short (not reaching the insertions of rostral setae), amost straight. One pair of porose areas *Ad* large, oval, located posterior to interlamellar setae.

*Notogaster*. Anterior notogastral margin weakly developed. Dorsophragmata (*D*) of medium size, elongate. Notogastral setae represented by 10 pairs of alveoli. Four pairs of porose areas round (*Aa*, *A3*, 18–20; *A1*, 16; *A2*, 10–12), with distinct borders. Alveoli of setae *la* inserted posteriorly to *Aa*. Lyrifissures *im* located anteriorly to *A1*. Opisthonotal gland openings (*gla*) poorly visible. Median pore represented as large alveolus, located in posterior part of notogaster between the virtual lines connecting *A2*–*A2*.

*Gnathosoma*. Morphology of subcapitulum, palps and chelicerae typical for most Galumnidae (for example: Ermilov and Anichkin 2010, 2011a, 2013b; Ermilov et al. 2011, 2013a). Subcapitular setae *h* (16) thin, smooth.

*Epimeral and lateral podosomal regions*. Apodemes (1, 2 sejugal, 3) well visible. Four pairs of epimeral setae observed ventrally; *1a*, *3b* (14–16) longer than *4a*, *4b* (10–12), all thin, smooth. Discidia triangular, circumpedal carinae distinct.

*Anogenital region*. Six pairs of genital (*g*_1_–*g*_2_, 14–16; *g*_3_–*g*_6_, 10–12), one pair of aggenital (14–16), two pairs of anal (14–16) and three pairs of adanal (14–16) setae thin, smooth. Anterior edge of genital plates with three setae. Adanal setae *ad*_3_ inserted laterally to adanal lyrifissures *iad.* Postanal porose area (*Ap*) rounded (18–20).

*Legs*. Three claws of each leg smooth. Morphology of leg segments, setae and solenidia typical for most Galumnidae (for example: Ermilov and Anichkin 2010, 2011a, 2013b; Ermilov et al. 2010, 2011). Formulae of leg setation and solenidia: I (1–4–3–4–20) [1–2–2], II (1–4–3–4–15) [1–1–2], III (1–2–1–3–15) [1–1–0], IV (1–2–2–3–12) [0–1–0]; homology of setae and solenidia indicated in Table 1.

**Figures 7–8.**
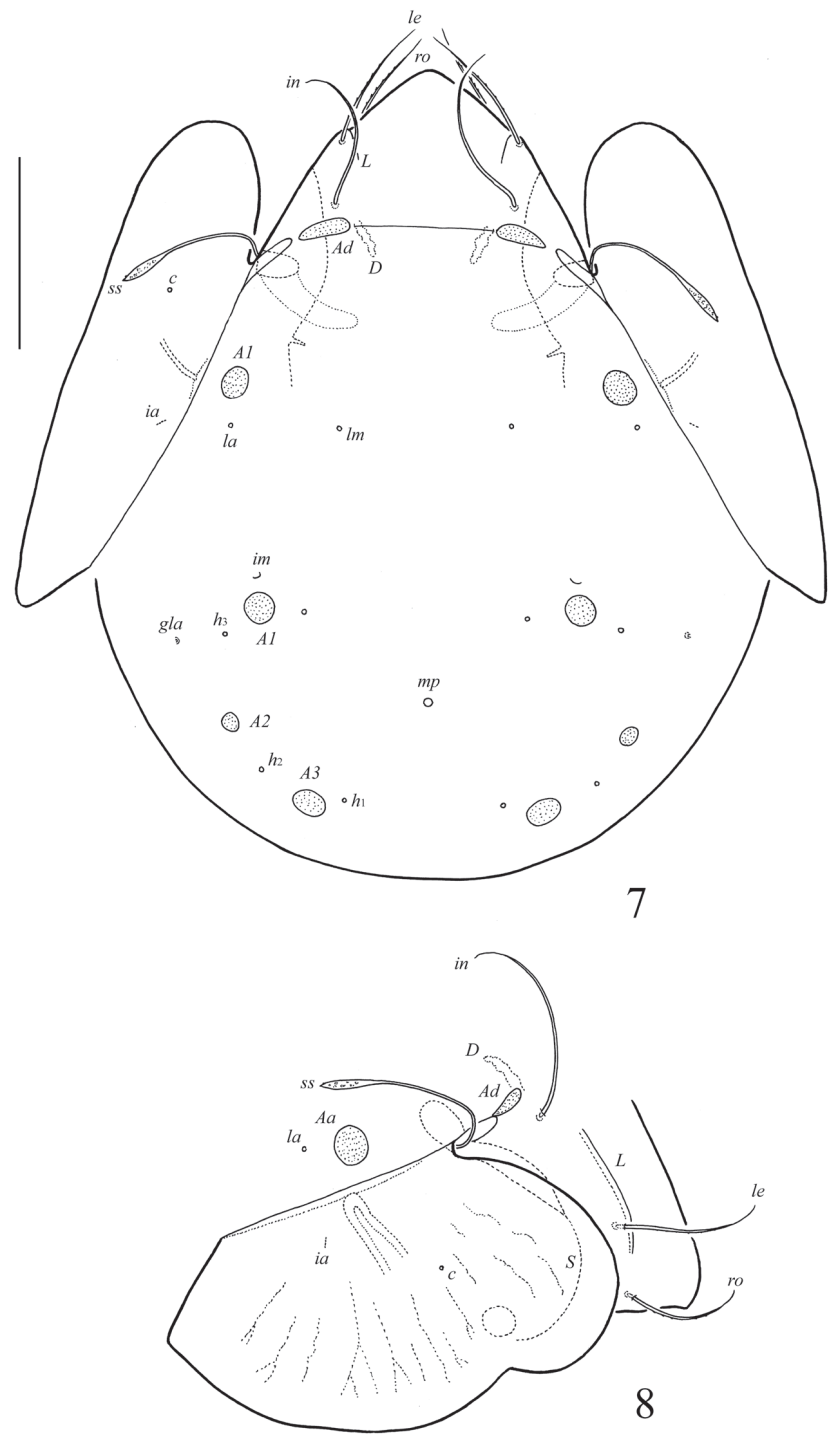
*Galumna (Galumna) paracalcicola* sp. n., adult: **7** dorsal view **8** dorso-lateral view of prodorsum, pteromorph and anterior part of notogaster. Scale bar 100 μm.

**Figures 9–10.**
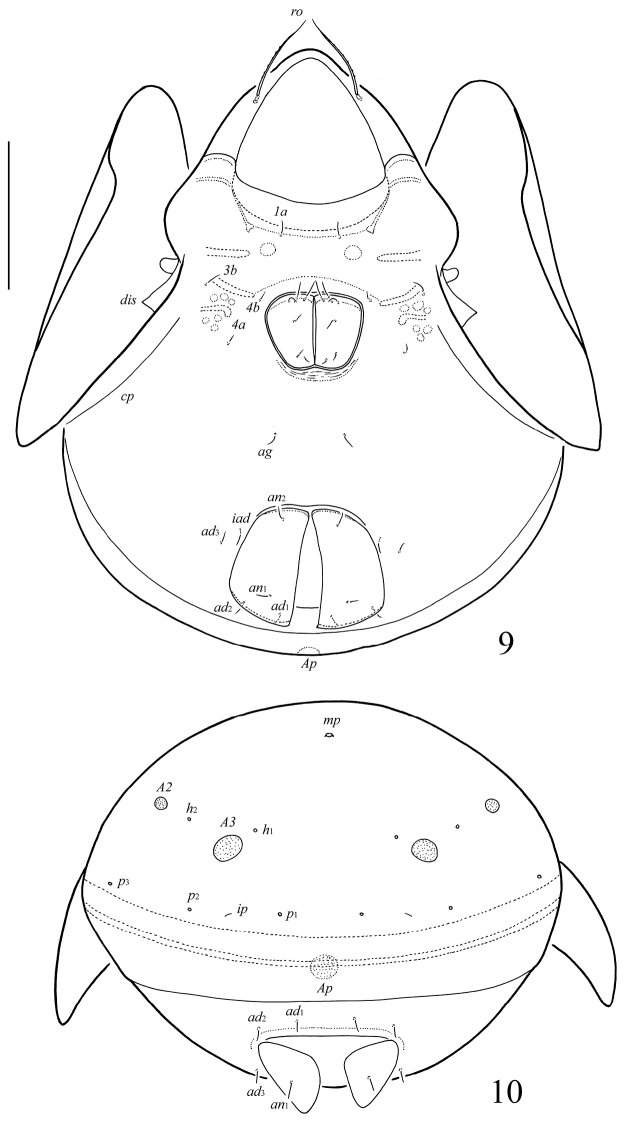
*Galumna (Galumna) paracalcicola* sp. n., adult: **9** ventral view (gnathosoma and legs not illustrated) **10** posterior view. Scale bar 100 μm.

#### Type deposition.

The holotype is deposited in the collection of the Zoological Institute of the Russian Academy of Sciences, St. Petersburg, Russia; paratype is deposited in the collection of the Tyumen State University Museum of Zoology, Tyumen, Russia.

#### Etymology.

The prefix *para* is Latin meaning “near” and refers the similarity between the new species and the species *Galumna calcicola* (Aoki & Hu, 1993).

#### Comparison.

*Galumna (Galumna) paracalcicola* sp. n. can be included in *calcicola*-group. Species of this group have the short (clearly not reaching the insertions of rostral setae), almost straight lamellar lines.

### Key to species of *calcicola*-group of the subgenus *Galumna (Galumna)*

**Table:** 

1	Porose areas *Aa* wedge-shaped or boot-shaped; anterior edge of genital plates with two setae	*Galumna (Galumna) lanceosensilla* Ermilov, Sidorchuk & Rybalov, 2011 (body size: 547–564 × 381–415; distribution: Ethiopia; see Ermilov et al. 2011)
–	Porose areas *Aa* rounded; anterior edge of genital plates with three setae	2
2	Sensilli setiform, with weakly dilated apical half; median pore absent	*Galumna (Galumna) calcicola* (Aoki & Hu, 1993) (body size: 284–288 × 220–227; distribution: southern China; see Aoki and Hu 1993)
–	Sensilli with well-developed lanceolate head; median pore present	*Galumna (Galumna) paracalcicola* sp. n. (body size: 398–415 × 298–332; distribution: Vietnam)

### Key to species of *Galumna (Galumna)* of Vietnam

**Table:** 

1	Rostrum pointed	2
–	Rostrum rounded	3
2	Lamellar lines straight, not parallel to sublamellar lines; rostral setae thickened, ciliate; porose areas *Aa* triangular	*Galumna (Galumna) kebangica* Ermilov & Vu, 2011 (body size: 547–581 × 381–415; distribution: Vietnam; see Ermilov and Vu 2012)
–	Lamellar lines curving backwards, parallel to sublamellar lines; rostral setae thin, slightly barbed; porose areas *Aa* rounded	*Galumna (Galumna) acutirostrum* Ermilov & Anichkin, 2010 (body size: 747–846 × 630–680; distribution: Vietnam; see Ermilov and Anichkin 2010)
3	Lamellar lines short (clearly not reaching the insertions of rostral setae)	*Galumna (Galumna) paracalcicola* sp. n. (body size: 398–415 × 298–332; distribution: Vietnam)
–	Lamellar lines long, reaching the insertions of rostral setae, or curving backwards, parallel to sublamellar lines	4
4	Interlamellar setae minute or represented by alveoli	5
–	Interlamellar setae well developed, long or medium size	8
5	Anterior margin of notogaster not developed; porose areas *A3* ribbon-shaped	*Galumna (Galumna) aba* Mahunka, 1989 (body size: 338–413 × 240–274; distribution: Vietnam; see Mahunka 1989)
–	Anterior margin of notogaster present; porose areas *A3* rounded or oval	6
6	Porose areas *Aa* boot-shaped; sensilli with weakly-developed, elongate head	*Galumna (Galumna) obvia* (Berlese, 1914) (body size: 705–898 × 584–647; distribution: semicosmopolitan; see Weigmann 2006; Bayartogtokh 2011; Ermilov et al. 2013a)
–	Porose areas *Aa* rounded, oval or triangular; sensilli clavate	7
7	Interlamellar setae represented by alveoli; sensillar head smooth	*Galumna (Galumna) levisensilla* Ermilov & Anichkin, 2010 (body size: 295–328 × 225–246; distribution: Vietnam; see Ermilov and Anichkin 2010)
–	Interlamellar setae minute; sensillar head ciliate	*Galumna (Galumna) flabellifera* Hammer, 1958 (body size: 303–348 × 204–220; distribution: Pantropic and Subtropic regions; see Hammer 1958; Aoki 1964, 1965, 1982)
8	Postanal porose area represented by one pair; lyrifissures *im* located latero-posteriorly to porose areas *A1*	*Galumna (Galumna) triquetra* Aoki, 1965 (body size: 469–540 × 327–342; distribution: Oriental region and Australia; see Aoki 1965)
–	Only single postanal porose area present; lyrifissures *im* located latero-anteriorly to porose areas *A1*	9
9	Sensilli setiform, without developed head	*Galumna (Galumna) pseudokhoii* Ermilov & Anichkin, 2011 (body size: 498–531 × 365–415; distribution: Vietnam; see Ermilov and Anichkin 2011b)
–	Sensilli with well-developed lanceolate head	*Galumna (Galumna) lanceata* (Oudemans, 1900) (? = *Galumna (Galumna) khoii* Mahunka, 1989) (body size: 528–670 × 363–460; distribution: Palaearctic region and Vietnam; see Pérez-Íñigo 1993; Weigmann 2006; Bayartogtokh 2011)

## Supplementary Material

XML Treatment for Allogalumna monodactyla

XML Treatment for Galumna (Galumna) paracalcicola
